# Risk Factors for Trauma-Related Eviscerations: Analysis of 821 Cases

**DOI:** 10.1155/2019/6198368

**Published:** 2019-11-11

**Authors:** Zhaoxin Jiang, Yao Yang, Yujie Li, Miner Yuan, Cheng Li, Xiaofeng Lin

**Affiliations:** State Key Laboratory of Ophthalmology, Zhongshan Ophthalmic Center, Sun Yat-sen University, Guangzhou 510060, China

## Abstract

Evisceration is the end therapeutic approach for the treatment of severe ocular trauma cases, and it is a tremendous loss for patients. In this study, we explored the changing trends in the number of surgeries performed, demographic data and ocular features, and risks for early evisceration, aiming to provide more data for the additional prevention and management strategies for this catastrophic problem. This was a retrospective study that included patients who underwent ocular evisceration at the Zhongshan Ophthalmic Center between January 2013 and December 2017. A total of 1229 evisceration cases were reviewed, and only trauma-related eviscerations were analyzed. Etiology, demographic data, ocular features, and hospitalization time were evaluated. The total number of trauma-related eviscerations recorded in the past five years was 821 cases. The number of surgeries performed was almost constant each year (164 ± 17 cases); 35% of the patients were less than ten years old at the time of the original ocular injury and 69% of them were male. Endophthalmitis led to significantly early evisceration compared with cases without endophthalmitis (*P* < 0.05). The group with a history of silicone oil tamponade showed a significantly longer surgical interval between trauma and evisceration than did the nonsilicone oil tamponade group (*P* < 0.05). Day-case hospitalization for evisceration increased from 0% to 32% over the past five years. The results of the present study show that the number of ocular trauma-related eviscerations performed in the past five years was almost unchanged and boys under ten are highly susceptible. This study also demonstrates that endophthalmitis leads to significantly early evisceration, and silicone oil tamponade may postpone ocular atrophy. Based on the study data, day-case surgery is safe for evisceration management.

## 1. Introduction

Evisceration is one of the end-stage therapies employed for the management of severe ocular diseases [1–3]. This surgery involves the complete evacuation of the intraocular contents, while the scleral shell and extraocular appendages are preserved. It plays an important role in clinical treatment. However, globe removal is a catastrophic loss to patients who lose both the functional use and the anatomical structure of the eye [4]. Thus, evisceration is one of the last options for both patients and doctors, with stringent indications.

Major indications of evisceration include a blind painful eye, endophthalmitis, phthisis bulbi, glaucoma, and severe traumatic injury [5–7]. Many studies, which aimed to improve the surgical outcome of evisceration, have been conducted on the exposure rate of orbital implants, long-term motility, and complication differences between porous and nonporous implants [8–10]. However, there is a scarcity of essential data on the changing trends in the number of surgeries performed, demographic and ocular features, risks for early evisceration, and utilization of day-case management for evisceration patients; analysis of these data is vital to enable possible reduction in the number of eviscerations performed and facilitate better management of evisceration patients.

Severe trauma is one of the main causes of evisceration [11, 12]. Analysis of the aforementioned factors may help to develop targeted prevention procedures to reduce the frequency of resorting to this devastating ocular treatment option. Thus, we conducted a retrospective analysis of the records of patients who underwent trauma-related evisceration from January 2013 to September 2017 in Zhongshan Ophthalmic Center, China. The aim of this study was to provide details of trauma-related evisceration cases, which we hope will increase interest and lead to the development of better management strategies for the prevention in this public health challenge.

## 2. Methods

This retrospective study was approved by the Sun Yat-sen University Medical Ethics Committee. The Zhongshan Ophthalmic Center, one of the largest tertiary eye care facilities in Guangzhou, China, provides eye care for the entire country. The clinical records of all patients who underwent evisceration from January 2013 to December 2017 at the Zhongshan Ophthalmic Center were retrospectively reviewed. The list of cases that underwent evisceration was supplied by the medical records management office of Zhongshan Ophthalmic Center. Cases with irregular data were not included. The number of eviscerations was recorded to show the trend of the changes in the number of surgeries performed in the past five years. Causes of evisceration were recorded for etiology analysis. Demographic data including age, sex, occupation, and injury-causing objects were reviewed. Ocular baseline data including visual acuity, intraocular pressure (IOP), and type and size of implants in the ocular socket were recorded. In addition, the duration of hospitalization was also analyzed.

Statistical analysis was performed using a commercially available statistical software package (SPSS for Windows, version 13.0, SPSS, Chicago, IL, USA). The data were expressed as mean ± standard deviation. The chi-squared nonparametric test was used to examine the impact of endophthalmitis and silicone oil tamponade on early evisceration. All *P*-values were two sided and values less than 0.05 were considered statistically significant.

## 3. Results

### 3.1. Major Reasons for Evisceration and Changes in the Number of Surgeries Performed over the Past Five Years

**Figure 1:**
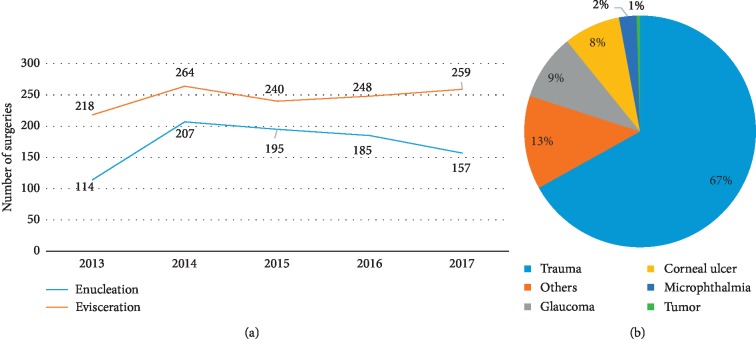
Number of eviscerations performed per year and major causes of evisceration. (a) Number of eviscerations and enucleations performed was 246 ± 18 and 286 ± 37 cases per year, respectively, between 2013 and 2017. (b) Trauma was the major cause (67%) for evisceration; glaucoma and corneal ulcer were responsible for 9% and 8% of the evisceration cases, respectively.

To enable better understanding of the overall situation of eviscerations, the total number of surgeries performed was recorded and analyzed (Figure 1); the total number of eviscerations performed over the past five years was 1129 cases. For changing trends in the number of eviscerations performed, the average number of surgeries was 246 ± 18 cases per year; the number of eviscerations performed each year was quite stable for the past five years, whereas the total number of inpatient surgeries performed in the same hospital rapidly increased in the past five years. Trauma was found to be the major reason for evisceration (67%), followed by glaucoma (9%) and corneal ulcer (8%). The total number of trauma-related eviscerations performed in the past five years was 821, with an average number of 164 ± 17 surgeries per year. Metal represented 25% of the material of the objects that caused trauma, followed by wood (12%). Further analysis of individual items showed that fireworks, knives, and grinding wheels represented 7%, 6%, and 4%, respectively.

### 3.2. Analysis of the Demographic Data of the Study Population

**Figure 2:**
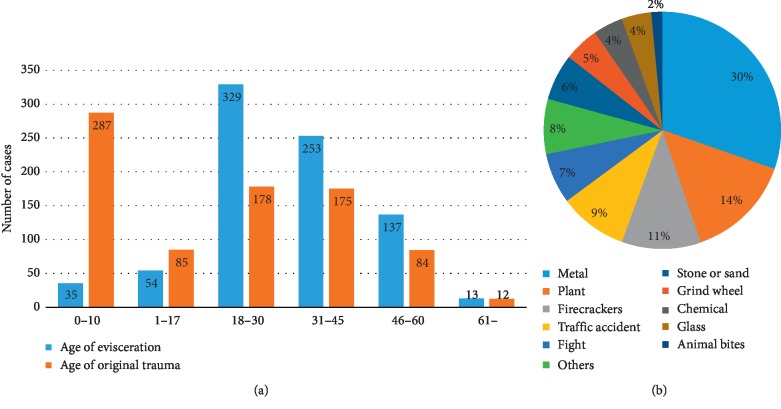
Analysis of the age distribution of the study population and of objects that caused their injury. (a) Most patients underwent evisceration when they were aged between 18 and 30 years, followed by the 31 to 45 years age-group; 89% of the patients who underwent evisceration were adults. However, when considering the age of the patient at the time of the original ocular trauma, most patients were under ten years old (35%), followed by 18–30 and 31–45 years old. (b) The materials of the objects that caused ocular trauma were mostly metal (25%), followed by wood materials (12%). Individual items that caused trauma included fireworks (11%) and grinding wheel (5%).

When analyzing demographic data, we found that 89% of the patients were adult (>18 years) and 40% of patients were 18 to 30 years of age (Figure 2). However, 45% of the patients were underage (<18 years) at the time of original ocular injury and 35% were under ten years of age when the injury occurred. With regard to sex, 79% of the total patients were male; 69% of the children under ten years of age were boys.

### 3.3. Analysis of the Baseline Ocular Features of the Evisceration Patients

Regarding visual acuity prior to surgery, 91% of the patients had a visual acuity of no light perception (NLP), whereas 7% had a visual acuity of light perception (Table 1). For intraocular pressure (IOP) analysis, ocular palpation was performed because the noncontact tonometer could not work for some patients who have an opaque cornea. There were 32% cases who were recorded as under Tn and 13% under 10 mmHg, whereas 21% recorded as Tn and 4% between 10 and 21 mmHg, and 17% cases recorded as higher than Tn. In all 822 evisceration cases, history of intraocular silicone oil tamponade was reviewed and only 47 cases (6%) had received this treatment. The time interval between ocular trauma and evisceration was significantly longer in the silicone oil tamponade group than in the nonsilicone oil tamponade group (5.97 ± 4.93 versus. 4.96 ± 11.06 years, *P* < 0.05).

Porous polyethylene implants (Medpor) were the most popular prostheses used for support of the ocular socket (75%), whereas hydroxyapatite orbital implants (Bio-Eye) were used in 25% of cases. For the diameter of the implants, 49% were 22 mm and 32% were 20 mm.

### 3.4. Relationship between Endophthalmitis and Evisceration

In all 821 evisceration cases, 88 endophthalmitis cases (11%) were diagnosed by histopathologic examination (Table 2). Among the endophthalmitis cases, 68 cases (78%) underwent evisceration in less than six months from the time of trauma, whereas this rate was only 33% in the nonendophthalmitis group (*P* < 0.05).

Next, pathogen analysis was performed via histopathologic investigation. Fungi were detected in 19 cases (11%), whereas 69 cases (89%) were suppurative endophthalmitis cases. Further data analysis showed that although pathogens were detected in all endophthalmitis cases, positive culture results were achieved in only 25 cases (25%).

### 3.5. Hospitalization Time and Day-Case Management in Evisceration Patients

**Figure 3:**
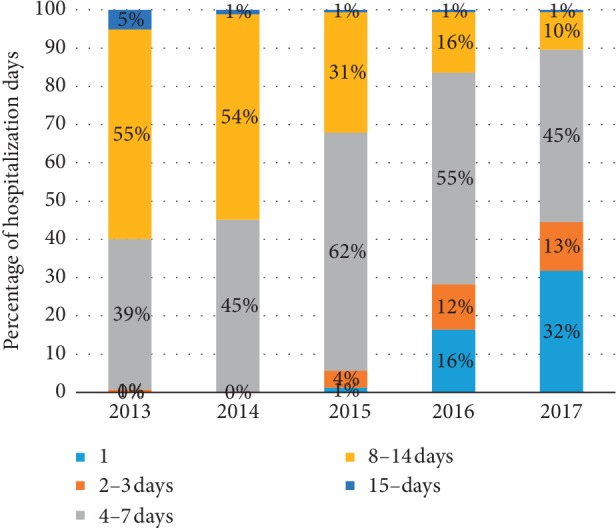
Trend of the changes in the number of days of hospitalization recorded at the study location over the past five years.

Day-case surgery means that patients check in and out of the hospital within 24 hours for a planned surgical procedure. This day-case management for evisceration, which was first set up in 2015 in Zhongshan Ophthalmic Center, resulted in an obvious decrease in hospitalization days in the past five years (Figure 3). In 2013 and 2014, none of the evisceration patients were hospitalized for less than two days, 40% of the patients stayed for three to seven days and more than 50% stayed for eight to 14 days. Since 2015, day-case hospitalizations increased from 1% to 32% in 2017. Meanwhile, the proportion of the 8- to 14-day hospitalizations decreased from 55% to 10% in the past five years. No adverse events directly related with day-case management were reported during this period.

In 2013 and 2014, none of the inpatients were hospitalized for less than two days, and more than 50% stayed for 8–14 days. Since 2015, day-case hospitalization increased from 1% to 32% in 2017, while the rate of 8–14-day-hospitalization decreased from 55% to 10% in the past five years.

## 4. Discussion

The results of the present study have shown that the total number of eviscerations performed per year has been quite stable over the past five years, and the proportion of trauma-related cases (67%) has also been stable. Cheng et al. reported 1375 enucleations from 2003 to 2006 [13], and Yoon et al. reported 802 anophthalmic surgeries from 1990 to 2005 [14], but no data were given as to the number of surgeries performed per year or regarding a change in the number of surgeries performed per year. The reason for the stability in the number of eviscerations performed is still unknown. According to our study data, a prediction of approximately 164 trauma-related eviscerations in the upcoming years may be accurate. Better prevention strategies need to be seriously considered.

More attention should be paid to ocular trauma prevention in children, especially boys. Data from the present study showed that males, especially 0- to 10-year-old boys, are the most susceptible population to evisceration. In the present study, the proportion of males who had ocular trauma and underwent evisceration was consistently higher than that of females; few studies have analyzed the proportion of sexes in adolescence with regard to ocular trauma and evisceration. For instance, Chaudhry et al. reported that 65% of males undergo evisceration, and Cheng et al. reported 81.3% of males undergo enucleation, but no data clearly showed the sex distribution in patients under 18 years [12, 13]. In the present study, males outnumbered females by 79% to 21% in all age-groups, and boys outnumbered girls by 69% to 31% for children under 10 years who underwent evisceration. Based on the data from the present study, a prevention system or education program needs to be set up in the early education stage, with special consideration on the psychology and praxeology of boys.

The associations among trauma, endophthalmitis, and evisceration are complicated. Studies have reported an incidence of 3.1% to 11.0% of endophthalmitis following open-globe trauma, and 14.3% of endophthalmitis cases end up in evisceration [15–17]. The present study provides additional data that endophthalmitis is a risk factor for early evisceration and accounts for 11% of trauma-related eviscerations. Lu et al. analyzed the risk factors for endophthalmitis cases that require evisceration or enucleation; their results showed that the female sex (43%), endogenous endophthalmitis, and delayed intervention were strongly associated with evisceration [18]. Tsai and Tseng reported that an older age, an NLP visual acuity, and corneal ulcer were associated with the need for evisceration [19]. However, the present study showed that most endophthalmitis-related evisceration patients were male (80%), relatively young (43 ± 15 years) and had NLP vision (75%).

In this retrospective study, patients who received intraocular silicone oil tamponade had a significantly longer interval before ocular atrophy. Nashed et al. reported on early surgical repair with silicone oil for 80 open-globe injuries; their results showed that 50% of patients retained ambulatory visual acuity during the 22-month follow-up [20]. We speculate that the support of silicone oil may help to restrain the shrinkage of the sclera and postpone ocular atrophy. One limitation of silicone oil application may be severe traumatic damage [21, 22]. In our previous studies, we designed a foldable capsular vitreous body (FCVB) as a novel artificial vitreous substitute [23–25]. Without keratectomy, the FCVB was implanted into the vitreous cavity after pars plana vitrectomy. Clinical trials have showed the safety and efficacy of using an FCVB for complicated retinal detachment, but the role of FCVBs as orbital implants is still being investigated [26–28].

One limitation of the present study is the lack of follow-up data. Complications including exposure rate and mobility scores were not recorded in the retrospective data used in this study. However, details on the incidence of implant exposure have been reported to range from 0–34% with varying follow-up durations [29–33]. The American Academy of Ophthalmology has compared the mobility and complications between porous and nonporous implants, and the results showed that both types are well tolerated and their complication rates are generally low [9].

## 5. Conclusion

The number of evisceration cases recorded per year has been stable for the past five years. Ocular trauma is the major cause of evisceration in this study, and our study data showed that boys under ten years are a highly susceptible population. Our analysis showed that endophthalmitis leads to significantly early evisceration, whereas silicone oil tamponade may postpone ocular atrophy. Day-case management works well for evisceration patients and hospitalization days decreased significantly in the past five years. Approximately 164 trauma-related eviscerations are projected to occur in the Zhongshan Ophthalmic Center in the coming year. Greater attention and better prevention strategies are highly required to curtail that occurrence of evisceration.

## Figures and Tables

**Table 1:** Demographic data and ocular features of the patients.

Survey item	Results			
Sex distribution of all age-groups	Male		Female	
n	651		170	
%	79		21	

Sex distribution of patients aged 0–10 years	Male		Female	
n	198		89	
%	69		31	

Visual acuity	NLP		>NLP	
n	747		74	
%	91		9	

Intraocular pressure	<Tn or <10 mmHg	Tn or 10–21 mmHg	>Tn or >21 mmHg	Undetectable
n	364	207	113	137
%	44	25	14	17

Interval	≤6 months		>6 months	
Silicone oil tamponade, n (%)	9 (1)		38 (4)	
Nonsilicone oil tamponade, n (%)	259 (32)		515 (63)	
			*P*=0.043	

The interval between ocular trauma and evisceration was significantly longer for patients who had silicone oil tamponade that for those who did not (5.97 ± 4.93 versus 4.96 ± 11.06 years, *P* < 0.05).

**Table 2:** Analysis of the relationship between endophthalmitis and evisceration.

Survey item	Results	
Endophthalmitis	Yes	No
n	88	733
%	11	89

Histopathologic investigation	Fungal endophthalmitis	Suppurative endophthalmitis
n	19	69
%	22	78

Pathogens culture	Yes	No
n	25	63
%	28	72

Interval	≤6 months	>6 months
Endophthalmitis, n (%)	68 (8)	20 (2)
Nonendophthalmitis, n (%)	204 (25)	529 (65)
		*P* < 0.001

Endophthalmitis cases showed significantly higher proportion of evisceration in less than six months (*P* < 0.05), indicating that endophthalmitis is a risk for early evisceration.

## Data Availability

All the data used to support the findings of this study are included within the article and are available from corresponding author by a reasonable request.
